# A Silent Threat: Exploring the Impact of Endocrine Disruption on Human Health

**DOI:** 10.3390/ijms24129790

**Published:** 2023-06-06

**Authors:** Yuet-Kin Leung

**Affiliations:** Department of Pharmacology and Toxicology, Winthrop P. Rockefeller Cancer Institute, University of Arkansas for Medical Sciences, Little Rock, AR 72205, USA; rickyleung@uams.edu

Endocrine-disrupting chemicals (EDCs) are chemicals, either natural or synthetic, that can interfere with the production, distribution, function, metabolism, or excretion of hormones in our body. Up to now, the EDCs that have been investigated are usually small molecules (less than 1000 Daltons) with one or more phenolic groups, and they share no specific structural similarity among them. However, because of their phenolic or polyaromatic ring structures, they are believed to mimic natural steroid hormones and allow them to interact with steroid hormone receptors and other extra-nuclear proteins/receptors.

EDCs are not the same as other toxic chemicals we studied in classical toxicology. EDCs can have different effects depending on the dose, the duration of exposure, and the individual’s susceptibility. They do not necessarily follow monotonic effects in the same way as other toxicants (e.g., a higher dose exerts more effect). Instead, they often show biphasic, multiphasic, or non-monotonic responses. Assumptions such as a lower dose of EDCs will cause less or no harm may not be applicable. Instead, a minimum dose of EDCs that could bind to a hormone receptor, which in turn interacts with downstream protein partners and triggers subsequent cellular events, will be enough to disturb any pre-programmed hormone effects. In some cases, this “low dose” could be as low as a physiological dose of a hormone, triggering receptor-mediated pathways. With higher doses, other activities such as chemical-induced oxidative stress become more pronounced to further disrupt other cellular activities.

The “low dose” effect is EDC-dependent. Regulatory organizations such as the US Food and Drug Administration (FDA) and the Environmental Protection Agency (EPA) define a reference or safe dose (RfD) of some EDCs based on the data collected in classical toxicology experiments. Is it really safe with this “low dose” (i.e., a dose lower than the safe dose)? Probably not. Take bisphenol A (BPA) as an example: based on the US FDA/EPA guidelines, the human RfD for BPA is 50 µg/kg/day. However, data from multiple experimental models showed BPA at a dose lower than RfD was associated with different signaling disruptions and heightened disease risks. It is puzzling why the US FDA still announced in 2022 that BPA is safe to use while another regulatory agency in Europe, the European Food Safety Authority (EFSA), further limits the use of BPA in 2023 and lowers the tolerable daily intake (TDI) level, which is used by the World Health Organization to estimate the safe level of exposure of a chemical, down to 0.2 ng/kg/day. We are still waiting to learn the argument behind the US FDA’s decision.

The effects of endocrine-disrupting chemicals (EDCs) are not routinely evaluated in current regulatory testing protocols. Despite this, humans are exposed to different forms of EDCs every day, through sources such as drinking water, food, personal care products, cooking utensils, and furniture. The effects of EDCs are often overlooked, as they may not be as readily detectable as those of carcinogens or toxicants. However, many EDCs are epigenetic modifiers, which can reprogram gene/protein expression spatially and temporally. The effects of EDCs can be particularly detrimental during windows of susceptibility/timing for critical growth and development, such as during the prenatal, perinatal, pubertal, or pregnancy period. EDCs can disrupt critical organs/tissues development, making them vulnerable to later life challenges from other environmental exposure, diet, or other lifestyle factors. Additionally, the epigenetic effects of EDCs can extend beyond exposed individuals to their unexposed generations (i.e., great-grandchildren). As we have only known about the topic of “endocrine disruption” for 25+ years, the transgenerational effect of EDCs in humans has not yet been shown, and most of the data observed thus far were collected from different experimental models. More research is therefore needed to understand the long-lasting effects of EDCs.

The International Journal of Molecular Sciences has released a Special Issue entitled *Endocrine Disruption and Human Diseases*, featuring eight contributions: three reviews and five original research articles providing recent updates on the field as well as novel experimental data to reveal how EDCs interfere with specific signaling and pathways, which could either lead to disease manifestation or augment disease risks.

Preterm birth (PTB) is defined as a birth that occurs before 37 weeks of gestation. PTB can be caused by a variety of factors, including maternal health problems, infections, and exposure to environmental toxins, such as EDCs. PTB can have serious consequences for both the mother and the child, including increased risk of infant mortality, respiratory problems, and developmental delays. Parturition is a process highly orchestrated by hormones, and disruption by EDCs could result in PTB. Drs. Hall and Kolan examined whether in utero exposure to EDCs, including BPA, phthalates, organochlorines, organophosphates, lead, and polybrominated diphenyl ethers (PBDE), is associated with PTB in their literature review [1]. The authors cited multiple cohort studies and discussed their associations with PTB. Although the findings are not all consistent, the authors identified a significant number of studies that demonstrate that exposure to EDCs is associated with an increase in PTB risk. The authors also highlight a few limitations of the studies and point out that “pesticides” could be the EDCs affecting most agriculture-based countries relating to the PTB problem.

Obesity can have serious consequences for both the health of the individual and the health of society, including an increased risk of heart disease, stroke, type 2 diabetes, and some types of cancer. Obesogens are chemicals that can cause weight gain through the disruption of lipid homeostasis and adipogenesis. To date, it has been discovered that obesogens can be EDCs acting through retinoid X receptors (RXRs) and peroxisome proliferator-activated receptor gamma (PPARγ). In the review written by Dr. Rato and his group [2], they discuss the disruption of energy metabolism as a mechanism of obesogens-mediated male infertility. In particular, they highlight nuclear receptors-associated signaling of tribuyltin (TBT), a well-studied obesogen, and review the role of obesogens in reprogramming the metabolism of Sertoli cells. The effects of obesogens/EDCs, including BPA, chlorpyrifos, lead, and polychlorinated biphenyls, on metabolism in reproductive tissues/cells of rodents are summarized. The authors also propose a few models, including in silico, in vitro, and ex vivo, for studying obesogen-related toxicity.

In another study conducted by Dr. Basak and his group [3], the authors investigated the outcomes of prenatal exposure to bisphenols (BPA and bisphenol S (BPS)) on sperm maturation and quality in PND90 rats. In this Special Issue, the authors reported that both BPA and BPS were associated with changes in the total fatty acid composition of the plasma and testis, but in a non-monotonic dose–response fashion. Consistent with other published data, as supported by this study, BPS, as a BPA substitute, is not necessarily safer or better than BPA. Using 4 μg/kg/day BPA as a single effective dose, the authors revealed that several enzymes in fatty acid anabolism and metabolism were impaired in the testis. In particular, the endogenous conversion of linoleic acid was impaired, and the expression of fatty acid desaturase 1 (FADS1) was reduced in the BPA group. The authors argued that such changes could affect spermatogenesis associated with bioenergetics, sperm motility, and its function. However, whether the changes reported in this study can impair sperm quality and motility or not requires further future follow-up studies.

EDCs are linked to cancer development, particularly in hormone-dependent cancers. In the review article written by Dr. de Assis [4], she and her team summarized the endocrine-disrupting activities of three common pesticides, including organochlorines, organophosphates, and carbamates reported on in the past, and discussed their associations with pediatric (including leukemia, Hodgkin’s and non-Hodgkin’s lymphoma, brain tumor, neuroblastoma, Ewing sarcoma, and Wilms’ Tumor, retinoblastoma) and adult cancers (including breast, vaginal/cervical cancer, melanoma, and endometrial cancer). The authors suggested epigenetics, including DNA methylation, histone modifications, and non-coding RNAs, as a potential mechanism of intergenerational and transgenerational transmission of pesticide-induced cancer susceptibility and referenced data from DDT/DDE, vinclozolin, methoxychlor, and persistent organic pollutants in multiple experimental models to support their hypothesis. Similar epigenetic inheritance through more than one generation has been observed in humans, and the authors discussed the Dutch Hunger Winter study as an example, which involved changes in DNA methylation as the key mechanism.

BPA has been shown to cause liver injury in animal studies. The mechanisms by which BPA may cause liver injury are not fully understood, but they may involve disruption of hormone signaling, inflammation, and oxidative stress. In this Special Issue, Dr. Tresguerres and his group evaluated whether BPA exposure during pregnancy would cause liver damage in dams and their PND6 female offspring [5]. They reported that perinatal exposure (up to PND6) to BPA at two doses showed an increase in oxidative stress, inflammatory response, and apoptosis in the livers of female rodents. It is noteworthy that the lower dose the authors used in this study was 36μg/kg/day, which is lower than the human US RfD but higher than the European TDI, and was shown to have stronger effects in the majority of the parameters measured when compared with the control group. Moreover, some of these parameters, including expression of antioxidant enzymes, lipid-DNA damage, oxidative stress, inflammation, and apoptotic markers measured in the PND6 female offspring showed non-monotonic u-shaped or inverted u-shaped responses. However, liver tissue damage was not yet observed in these offspring.

EDCs have been shown to affect the neuronal system in both animals and humans. Using novel bioinformatics analyses, Dr. Hrelia and her team published a study in this Special Issue [6] and revealed that exposure of a neuroblastoma cell line, SH-SY5Y, to subtoxic concentrations of vinclozolin (VNZ) dysregulated microRNA (miR-29-3p) expression as well as perturbed the expression of its downstream ADAM12 and CDK6, which may, in turn, activate the PI3K/Akt/mTOR signaling pathway and suppress tumor suppressor p53 level. This observation was specific to VNZ as the authors compared the effects with two other EDCs, atrazine and cypermethrin, which are commonly used in the agriculture industry. Some of the data suggested that the lower dose of VNZ showed more pronounced effects than the higher dose. Future research is needed to investigate the relationship between neuronal carcinogenesis and exposure to low doses of VNZ using in vivo models.

Chemotherapy resistance is a major issue in many types of cancer, especially for those cancers with limited therapeutic options, e.g., ovarian cancer. In this Special Issue, Dr. Rizvi and his team evaluated the effects of exposure to per- and poly-fluoroalkyl substances (PFAS) mixture on the survival of two carboplatin-treated human ovarian cancer cell lines (from high-grade serous carcinoma, OVCAR-3 and Caov-3) [7]. The authors revealed that the sub-cytotoxic dose of select PFAS and its mixture was pro-survival, even in the presence of carboplatin. This suggests that exposure to certain PFAS/PFAS mixtures could make a specific chemotherapy treatment (i.e., carboplatin in this study) ineffective, leading to the problems of drug resistance. Such pro-survival effects with the PFAS treatment were associated with an increase in mitochondrial potential. Since PFAS persist in the environment, this could provide some new insights into why certain cancer therapies fail for certain patients.

The early stage of prostate cancer is a hormone-dependent disease. However, how EDCs augment prostate cancer risk is still currently under investigation. In the article published by Dr. Deoraj’s team [8], they utilized publicly available data from the National Health and Nutrition Examination Survey (NHANES) and examined the association between environmental exposure to phenols (BPA, Benzophenone-3,4-tert-octylphenol, and triclosan) and parabens (butylparaben, ethylparaben, methylparaben, and propylparaben) and prostate cancer incidence. They further pulled gene information associated with each EDC using the Comparative Toxicogenomic Database and conducted bioinformatics gene ontology and pathway analyses and found a subset of genes that were common to all EDCs studied. Five hub genes (BUB1B, TOP2A, UBE2C, RRM2, and CENPF) were found to be highly associated with the aggressive stage/pathological grade of prostate cancer using The Cancer Genome Atlas data. Further investigation using experimental models may warrant the development of novel diagnostic markers and therapeutic drug targets.

In this Special Issue, we cover studies on EDCs ranging from in vitro to in vivo as well as human epidemiology studies, and discuss the underlying mechanisms from rapid signaling in a cell to epigenetic changes in multiple generations. Only one study touched on the topic of non-monotonic dose responses, and several studies investigated the effects of EDCs at subtoxic or even low doses. For future EDC studies, we should systematically analyze the dose response of each EDC and EDC mixture to understand their impact on human health. We should also include any biological or molecular endpoints beyond exposure windows or even more than one generation in certain EDC evaluation protocols. Additionally, we should conduct high-throughput multi-omics analyses, which will allow us to determine disease risks prior to any disease development.
